# Role of Gut Bacteria in Enhancing Host Adaptation of *Tuta absoluta* to Different Host Plants

**DOI:** 10.3390/insects15100795

**Published:** 2024-10-13

**Authors:** Luo-Hua Shang, Xiang-Yun Cai, Xiu-Jie Li, Yu-Zhou Wang, Jin-Da Wang, You-Ming Hou

**Affiliations:** 1 State Key Laboratory of Ecological Pest Control for Fujian and Taiwan Crops, Ministerial and Provincial Joint Innovation Centre for Safety Production of Cross-Strait Crops, Fujian Agriculture and Forestry University, Fuzhou 350002, China; shanglh215@163.com (L.-H.S.); 15797686945@163.com (X.-Y.C.); 19861909770@163.com (X.-J.L.); 18558912999@163.com (Y.-Z.W.); 2 National Engineering Research Center of Sugarcane, Fujian Agriculture and Forestry University, Fuzhou 350002, China

**Keywords:** insect gut bacteria, SMRT, bacterial biomarker, *Tuta absoluta*, host adaptability

## Abstract

**Simple Summary:**

Insect gut commensal bacteria play an important role in insect adaptation to host plants. Feeding host plants is the main reason affecting the biomarker gut bacteria of insects, and it is of great significance to screen out a bacterial biomarker that can be cultured in vitro for insect host adaptability and pest control. There were few reports on the adaptability of host plants mediated by gut bacteria to *Tuta absoluta*. In this study, the bacterial biomarkers were screened and cultured from the growth and development analysis of tomato and potato. It was found that the biomarker bacteria of different strains of *T. absoluta* were helpful in improving the adaptability of *T. absoluta* to the original host plants and laying a foundation for the study of the interaction mechanism between insects and plants mediated by gut bacteria.

**Abstract:**

The insect gut bacteria play important roles in insect development and growth, such as immune defense, nutrient metabolism, regulating insect adaptations for plants, etc. The *Tuta absoluta* (Meyrick) is a destructive invasive pest that mainly feeds on solanaceae plants. However, the relationship between gut microflora and host adaption of *T. absoluta* remains to be known. In this study, we first compared the survival adaptability of *T. absoluta* feeding with two host plants (tomatoes and potatoes). The *T. absoluta* completed the generation cycle by feeding on the leaves of both plants. However, the larvae feeding on tomato leaves have shorter larvae durations, longer adult durations, and a greater number of egg production per female. After Single Molecular Real-Time (SMRT) sequencing, according to the LDA Effect Size (LEfSe) analysis, the gut bacterial biomarker of *T. absoluta* fed on tomato was *Enterobacter cloacae* and the gut bacterial biomarker of *T. absoluta* fed on potatoes was *Staphylococcus gallinarum* and *Enterococcus gallinarum*. Furthermore, a total of 6 and 7 culturable bacteria were isolated from the guts of tomato- and potato-treated *T. absoluta*, respectively. However, the isolated strains included bacterial biomarkers *E. cloacae* and *S. gallinarum* but not *E. gallinarum*. In addition, different stains bacterial biomarkers on *T. absoluta* feeding selection were also studied. *E. cloacae* enhanced the host preference of the SLTA (*T. absoluta* of tomato strain) for tomato but had no impact on STTA (*T. absoluta* of potato strain). *S. gallinarum* improved the host preference of STTA to a potato but did not affect SLTA. The results showed that the gut bacteria of *T. absoluta* were affected by exposure to different host plants, and the bacterial biomarkers played an important role in host adaptability. This study not only deepens our understanding of gut bacteria-mediated insect-plant interactions but also provides theoretical support for the development of environmentally friendly and effective agricultural pest control methods.

## 1. Introduction

Herbivorous insects have formed complex interactions with host plants during long-term evolution. In order to cope with the changes in living environment, insects have evolved the ability to adapt to a variety of host plants, such as *Spodoptera litura*, *Spodoptera frugiperda*, *Tuta absoluta,* and so on [1,2,3]. This adaptive ability not only allows polyphagous insects to obtain the nutrients necessary for development and reproduction but also acquires the ability to metabolize, digest, and detoxify different host plants [4,5,6,7]. It has been reported that the adaptation mechanism of herbivorous insects to different host plants mainly includes: (1) insects regulate their physiological and behavioral changes to adapt to plants; for example, *Mythimna separata* caterpillars adjust the body’s intake of protein and carbohydrates according to the nutritional content of the host and prepares for the subsequent development stage [8]. Besides, the recognition of plant or chemical volatiles by the insect olfactory system is helpful for them to select and adapt to hosts [9]. For instance, the antennal binding protein-X (ABPX) and general odorant-binding protein-2 (GOBP2) of *Agrotis ipsilon* play a role in crop adaptation and environmental recognition [10]. *Spodoptera littoralis* can select adaptive host plants from a variety of host plants by using olfaction [11]. (2) Insects use the high expression of gut detoxification metabolic enzymes, digestive protease enzymes, and related genes to improve their host adaptability; for instance, *S. frugiperda* promoted its adaptability to non-preferred host rice by increasing the expression of Cytochrome p450s genes [12,13]; The changes of digestive enzyme-related genes in *Frankliniella occidentalis* were related to the differences in their adaptation to different host plants [14]; What’s more, digestive physiology mediates the adaptation of *Hyphantria cunea* larvae to multiple host plants [15]. (3) Insect gut bacteria degrade plant toxins, secondary metabolites, etc., to assist insects in quickly adapting to host plants. Gut bacteria could enhance the adaptability of *H. cunea* to non-preferred host plants [16]. The Middle East Asia Minor 1 species of *Bemisia tabaci* used different dominant bacteria to improve its adaptability to different host plants [17]: *Enterobacter* sp. EbPXG5 in the gut biota of *Plutella xylostella* degraded the secondary metabolites of plant kaempferol, thereby enhancing the ability of *P. xylostella* to adapt to host plants [18].

The insect gut bacteria play a role in helping insects digest and detoxify the plants they ingest. According to reports, insect gut bacteria, a subset of insect endosymbionts residing in the insect gut, play a critical role in various biological processes within the host organism. These processes include the regulation of nutrient metabolism, facilitation of food digestion, promotion of growth and development, enhancement of fecundity, influence on population dynamics, modulation of insect adaptability to environmental and plant factors, and bolstering of insect defense mechanisms [19,20,21]. The maintenance of gut flora homeostasis is essential in this context [22]. Insect gut bacteria promote the growth and development of insects. By feeding the *Musca domestica* larvae with *Enterobacter hormaechei*, their body weight and body length increased significantly, and the growth cycle was significantly shortened, suggesting that *E. hormaechei* promoted the growth and development of *M. domestica* larvae [23]. The weight of *Hermetia illucens* larvae fed with *Rhodococcus rhodochrous* was significantly higher than that without *R. rhodochrous* [24]. Antibiotic treatment reduced the survival rate of *Nilaparvata lugens*; conversely, the supplementation of *Bacillus tequilensis* (S36) has been shown to significantly restore the survival rate of *N. lugens* [25]. In summary, by analyzing the changes in insect weight, growth duration survival rate, and other physiological indexes of insects after feeding on plants treated with gut bacteria, it is shown that gut bacteria can change the adaptability of insects to host plants. Host plants serve as the primary reservoir of insect gut bacteria, as evidenced by research indicating that bacteria present in the ingested plant material are introduced into the insect gut upon feeding. Subsequently, some of these bacteria are able to establish colonization within the insect gut tract, while the remainder are expelled through excretion [26,27]. The gut is the main place for plants to be digested by herbivorous insects, and gut microorganisms also play an important role in insect digestion. Due to the different types of gut bacteria involved in the digestion or defense of different hosts, the gut flora of insects will change when insects long-term feed on sample host plants. Li et al. found that *Sitobion miscanthi* was infected with *Hamiltonella defensa* in the gut after feeding on plant leaves infected with *H. defensa* [28]. Pons et al. demonstrated that *Serratia* symbiotica colonizes the intestines of aphids following the aphids’ plant juice containing *S.* symbiotica [29]. Zhang et al. showed that the richness and diversity of bacterial species in the gut of the fifth instar larvae of *Rhoptroceros cyatheae* feeding on *Gymnosphaera metteniana* were slightly higher than those of *Alsophila spinulosa* [30]. The gut-dominant bacteria of insects fed on plants were different from those fed on artificial diets [31,32]. In Lepidoptera insects, Deng et al. observed that the abundance and diversity of gut bacteria in the fourth instar larvae of *H. cunea* fed on *Platanus orientalis* were higher than those fed on artificial diets, indicating that different foods could affect the gut bacterial diversity of *H. cunea* larvae [33]. In addition, the change in *H. cunea* gut flora can improve its adaptability to new host plants [16]. The gut bacteria of both *Grapholita molesta* and *Spodoptera frugiperda* were affected by host plant species and played an important role in insect-host plant adaptation [34,35]

*T. absoluta* is a major invasive pest of Gelechiidae, Lepidoptera. It mainly damages solanaceae plants, especially tomatoes (including fresh tomatoes, processed tomatoes, and cherry tomatoes), potatoes, eggplants, etc., which mainly harms host plants during the larval stage and poses a serious threat to agricultural production [36,37]. Previous 16SrRNA/16SrDNA sequencing only analyzed gut bacteria at the genus level, while SMRT sequencing could analyze and identify the species level of gut bacteria, which is convenient for our subsequent research on the function of single species [38,39]. SMRT (SMRT Link, version 8.0) has the characteristics of long sequence reading and higher accuracy in identifying strains. It is also known as the third-generation sequencing technology and was based on the PacBio sequencing platform to sequence the DNA sequence encoding the 16S rRNA region [40,41]. Given the pivotal role of host plants in shaping the gut flora of phytophagous insects and the significance of gut bacteria in host insects, we conducted a comprehensive analysis of the growth and development of *T. absoluta* fed on tomato and potato, as well as the composition and abundance of gut bacteria. This was followed by the isolation, culture, identification, and functional analysis of the bacterial biomarker so as to deeply analyze the mechanism of gut bacteria-mediated plant-insect interaction and provide a theoretical basis for the green control of agricultural pests.

## 2. Materials and Methods

### 2.1. Insects and Plants

Tomato (*Solanum lycopersicum*, Zhefen202) and potato (*Solanum tuberosum*, Helan15) were planted in the experimental field of Fujian Agriculture and Forestry University. *T. absoluta*, collected from Chuxiong, Yunnan, China. They were reared in an incubator with a temperature of 25 ± 1 °C, relative humidity of 65 ± 5%, and photoperiod Light:Dark = 16:8.

### 2.2. Bioassay of T. absoluta

Tomato and potato leaves were used to feed the newly hatched larvae of *T. absoluta*, which were divided into two groups: the tomato strain *T. absoluta* (SLTA) and the potato strain *T. absoluta* (STTA). Fifty larvae in each group were fed individually and observed daily. Fresh plant leaves were replaced every 24 h. The egg duration, larvae duration, adult duration, egg production per female, pupation rate, and eclosion rate of the third generation of *T. absoluta* in the two groups were counted. The experimental data were statistically analyzed using SPSS 26.0 software and plotted using GraphPad Prism 9.

### 2.3. Gut Samples Collection of T. absoluta

Before the gut anatomy of *T. absoluta*, these larvae were first disinfected with 75% alcohol in an ultra-clean bench, then rinsed with sterile water twice, and the gut was taken out with sterile tweezers and placed in a 1.5 mL sterile centrifuge tube containing 200 μL PBS buffer. After the samples were collected, they were quickly frozen in liquid nitrogen and stored at −80 °C. Three replicates were set up for the *T. absoluta* treated with tomato (SLTA1-3), and the potato feeding treatment *T. absoluta* was set to three replicates for STTA1-3, and the guts of 50 fourth instar larvae were collected for each replicate. Finally, the samples were sent to Biomarker Technologies Co., Ltd. (Beijing, China) for SMRT sequencing.

### 2.4. Establish Library and SMRT Sequencing of Gut Bacteria

The total DNA of six samples was extracted by using the TGuide S96 Magnetic bead genomic DNA extraction kit (TIANGEN, Cat. No. DP812), and primers with Barcode were synthesized for PCR amplification. After purification, quantification, and homogenization of the PCR products, the SMRT Bell sequencing library was formed. The qualified library was sequenced by PacBio Sequel Ⅱ, and the CCS (Circular Consensus Sequencing) file was exported for subsequent data analysis. The original subreads were corrected to get the CCS sequence, followed by lima (v1.7.0) software to identify CCS sequences of different samples by barcode sequence and remove chimeras, high-quality CCS sequences (16S: 1200–1650 bp) are obtained. The Usearch (version 10.0) software was used to cluster the Operational Taxonomic Units (OTUs) at the level of 97% similarity, and 0.005% of the sequenced sequences were used as the threshold to filter the OTUs. The analysis process mainly included sequencing data quality assessment, gut bacteria α and β diversity analysis, composition structure and abundance analysis, gut bacteria difference analysis of different host treatments, and bacterial function prediction analysis. The α diversity reflects the richness and diversity of species within a single sample; the α was analyzed using QIME2 software (https://qiime2.org/, accessed on 20 November 2023). Among them, Ace and Chao1 count for species richness. Simpson and Shannon index reflects species diversity. The β diversity analysis was processed by Quantitative Insights into Microbial Ecology (QIIME2 2020) software to compare species diversity between different samples, and the Bray–Curtis statistical algorithm was used to calculate the distance between samples. The test was carried out by permutational multivariate analysis of variance (PERMANOVA), and the confidence level was 95%. The β diversity analysis mainly includes: Principal coordinates analysis (PCoA), Non-metric multi-dimensional scaling (NMDS) analysis, and the Unweighted Pair-group Method with Arithmetic Mean Analysis (UPGMA). Based on the SILVA ribosomal RNA database (http://www.arb-silva.de, accessed on 20 November 2023), the abundance of gut bacteria of *T. absoluta* treated with different host plants at different classification levels was analyzed by using the naive Bayesian classifier combined with the comparison method, and then the community structure map was drawn by using BMKCloud (http://www.biocloud.net, accessed on 20 November 2023). The results showed that the abundance of gut bacteria of *T. absoluta* treated with different host plants was significantly different at different classification levels. Line Discriminant Analysis Effect Size (LEfSe, https://huttenhower.sph.harvard.edu/lefse/, accessed on 20 November 2023) used linear discriminant analysis (LDA) to estimate the effect of the abundance of gut bacteria on the difference between SLTA and STTA, according to the LDA > 4 and the *p* < 0.05 to determine whether the bacteria belong to the marker species, also known as the bacterial biomarker [42]. All data analysis and graphic drawing were performed using the Biocloud platform (https://www.biocloud.net, accessed on 20 November 2023). The raw data of SMRT sequencing has been uploaded to the NCBI Sequence Read Archive (SRA) database with accession number PRJNA1137249.

### 2.5. Isolation and Identification of Culturable Gut Bacteria

The prepared gut homogenate of *T. absoluta* was diluted to 1.0 × 10^−1^, 1.0 × 10^−2^, 1.0 × 10^−3^, 1.0 × 10^−,4^ and 1.0 × 10^−5^, and 100 μL of diluent was drawn and coated on nutritious agar (NA) medium respectively, each concentration was repeated three times. The cells were cultured in the dark at 37 °C and relative humidity (70 ± 5)% for 24 h. The number of colonies with different colors, shapes, and sizes on each medium was recorded, and single colonies with different shapes were selected and purified 2–3 times on NA medium and stored at 4 °C refrigerator. According to ‘Bergey’s Manual of Determinative Bacteriology’ [43], the size, shape, color, gloss, transparency, and uplift state of the isolated single colonies were observed and recorded. The isolated strains were stained by Gram’s staining method, and the cell morphology and Gram’s negative and positive bacteria were observed under an oil microscope [44,45].

The bacterial suspension of a single colony was used as a bacterial PCR template. The bacterial identification universal primers 27 forward primers (5′-AGAGTTTGATCCTGGCTCAG-3′) and 1492 reverse primer (5′-CGGTTACCTTGTTACGTTACGACTT-3′) [46]. The PCR reaction system includes 2 × Hieff Canace^®^ Gold PCR Master Mix (10 μL), forward primer (1 μL), reverse primer (1 μL), bacterial liquid (2 μL) and ddH_2_O (6 μL). The PCR reaction conditions were initial denaturation at 94 °C for 3 min, and the following 29 cycles were 94 °C denaturation for 30 s, 48 °C annealing for 30 s, 72 °C extension for 2.5 min, and 72 °C final extension for 5 min. The PCR products were detected by 1% agarose gel electrophoresis with 5 μL PCR products, and the qualified PCR products were sent to the company of Sangon Biotech for sequencing. The sequencing results were spliced and compared by ContigExpress software (version 6.0), and the strains with the highest similarity were found in NCBI (National Center for Biotechnology Information (nih.gov), accessed on 20 November 2023), and the sequences were saved. The phylogenetic tree was constructed by using MEGA 11.0 software, Neighbour-joining, and Kimura two-parameter correction model.

### 2.6. Feeding Selection of Different Strains of T. absoluta on Two Bacterial Biomarkers

The single colonies of the two biomarker strains, *Enterobacter cloacae* (*Enterobacter cloacae* strain TAGSL1, PQ044797) and *Staphylococcus gallinarum*, (*Staphylococcus gallinarum* strain TAST21, PQ045250), were selected and inoculated into 1 mL liquid Nutrient Broth (NB), respectively, and cultured in a shaker at 37 °C and 200 rpm for 18 h. After that, 100 μL bacterial solution was inoculated into 15 mL liquid NB for amplification. After re-incubation for 18 h, the bacteria were centrifuged at 5000× *g* for 5 min to collect. The bacteria were washed with sterile water and centrifuged 3 times. The bacterial solution with an optical density (OD) of 1 was prepared with sterile water for later use.

The fresh tomato leaves (SL) and potato leaves (ST) were completely immersed in two kinds of bacterial solution for 5 h and dried on the benchtop, recorded as SL + *E*. *cloacae* SL + *S*. *gallinarum*, ST + *E*. *cloacae*, ST + *S*. *gallinarum*, respectively. The leaves treated with sterile water were used as the control. The leaves treated with two kinds of bacterial solution and sterile water were symmetrically placed at both ends of a circular petri dish with a diameter of 15 cm. Fifteen 2–3 instar larvae of *T. absoluta* treated with starvation for 2 h were placed in the center of the petri dish. Each treatment was repeated three times, and the number of larvae on the leaves of different treatments after 12 h was photographed and counted.

## 3. Results

### 3.1. Analysis of the Growth and Development of T. absoluta on Two Host Plants

**Figure 1:**
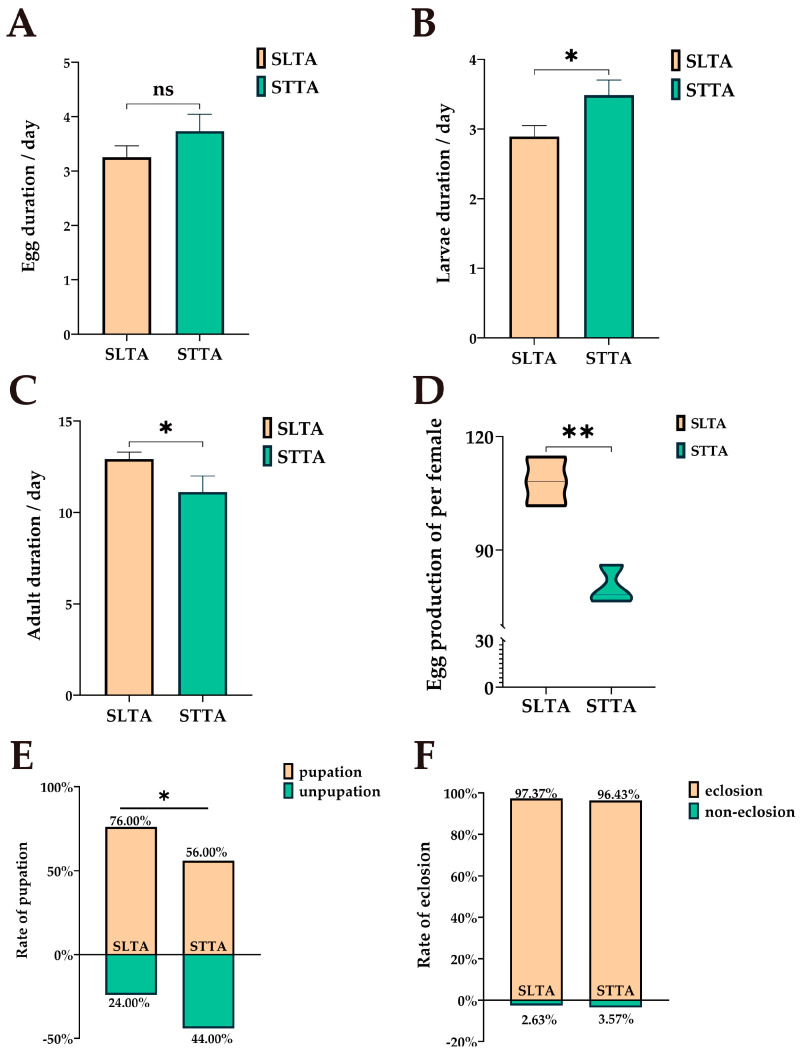
Comparison of the growth and development of *T. absoluta* fed on two hosts: (**A**) Egg duration; (**B**) Larvae duration; (**C**) Adult duration; (**D**) Egg production per female; (**E**) Pupation rate; (**F**) Eclosion rate; SLTA means *T. absoluta* feeding on tomatoes; STTA means *T. absoluta* feeding on potatoes. The difference is based on an independent sample *t*-test, “ns” represents no difference; “*” represents *p* < 0.05; “**” represents *p* < 0.01.

Two host plants, tomato and potato, had significant effects on the developmental duration of *T. absoluta* (Figure 1A–C). There was no significant difference in the developmental duration of *T. absoluta* eggs between the two groups (*p* = 0.134). However, there is a significant difference between larvae (*p* = 0.012) and adults (*p* = 0.025): The larval duration of SLTA was shorter than that of STTA, indicating that the larvae feeding on tomato could obtain sufficient nutrition in a short time to enter the next period (Figure 1B); The adult duration of SLTA was longer than that of STTA, the results showed that the adults of *Tuta absoluta* feeding on tomato had longer oviposition period and greater reproductive potential (Figure 1C); At the same time, the egg production (*p* = 0.004) and pupation rate (*p* = 0.019) of group SLTA were significantly higher than those of group STTA, which once again proved that feeding tomato was more adaptable than feeding potato (Figure 1D,E). This conclusion is the same as the traditional hypothesis under the experimental conditions, that is, the adaptation effect of *Tuta absoluta* on tomato is better than that of potato.

### 3.2. Analysis of Gut Bacterial Diversity of T. absoluta Based on SMRT

A total of 205,242 Raw Circular Consensus Sequencing (CCS) were obtained by SMRT sequencing of gut 6 samples of *T. absoluta* fed with tomato and potato, with an average of 34,207 CCS sequences per sample. After Barcode recognition of Clean CCS sequences, length filtering, and chimera removal, a total of 203,013 Effective CCS sequences were obtained (Table S1). The Effective CCS sequence was divided into 63 OTUs, and there are 34 OTUs in SLTA and STTA, 10 proprietary OTUs in group SLTA, and 19 unique OTUs in group STTA. This shows that the division of OTUs in the gut flora of the *T. absoluta* is affected by the treatment of different host plants, and the number of OTUs in the gut flora of the non-adaptive host plant (potato) treatment group is more (Figure S1).

**Figure 2:**
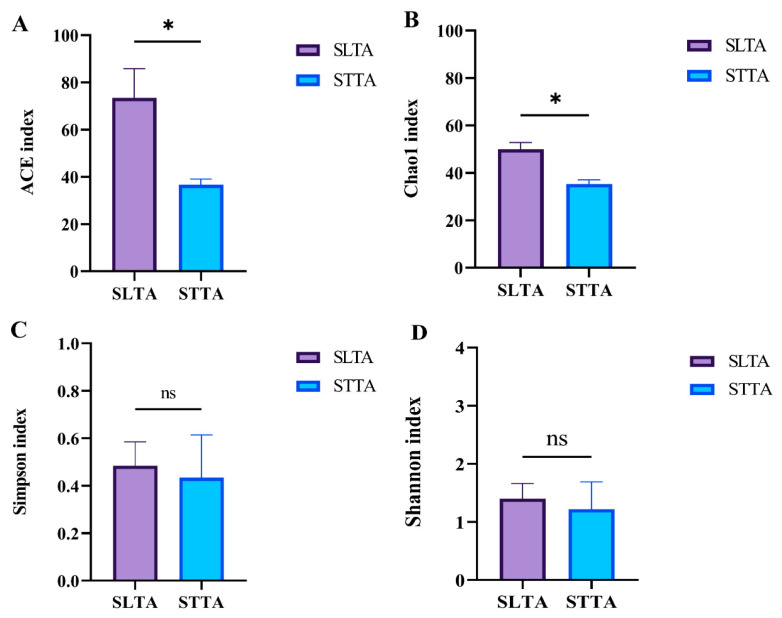
Analysis of the difference of α diversity index of gut bacteria of SLTA and STTA: (**A**) was ACE index; (**B**) was Chao1 index; (**C**) was Simpson index; (**D**) was Shannon index; Data analysis was performed using an independent sample *t*-test, “ns” represents no difference; “*” represents *p* < 0.05.

The α diversity analysis of gut bacteria of *T. absoluta* treated with two host plants showed that the ACE index (*p* = 0.043) and Chao1 (*p* = 0.011) index of the tomato feeding treatment group were significantly higher than those of the potato group, indicating that the gut bacteria richness (Number of bacteria) of *T. absoluta* fed with tomato was higher (Figure 2A,B). There was no significant difference in Simpson index (*p* = 0.819) and Shannon index (*p* = 0.746), indicating that there was no significant difference in the diversity of gut flora between the two groups of plants (Figure 2C,D). It indicated that the number of gut bacteria was different after different hosts treated *T. absoluta*, but had no effect on the diversity of bacteria.

The analysis of gut bacterial β diversity between tomato group and potato group showed that there were differences in bacterial community structure between the two groups. PCoA (*p* = 0.001, R^2^ = 0.67; Figure S2A) and NMDS analysis (Stress = 0.000, *p* = 0.001, R^2^ = 0.67; Figure S2B) all showed that the similarity of bacterial communities between the two groups was significantly different. Meanwhile, The UPGMA results showed that the bacterial communities of the two groups were divided into two categories. The bacterial communities of the tomato group were in one category, and the bacterial communities of the potato group were in the other category, indicating that there were differences in the similarity of bacterial community structures between the two groups. At the family level, the dominant bacteria family in SLTA was *Enterobacteriaceae,* and the dominant bacteria family in STTA was *Enterococcaceae* (Figure S3C). At the genus level, the dominant bacteria genus in SLTA was *Enterobacter,* and the dominant bacteria genus in STTA was *Enterococcus* (Figure S3D). The dominant bacteria in the tomato treatment group were *Enterobacter cloacae,* and the dominant bacteria in the potato treatment group were *Enterococcus gallinarum* (Figure S3E).

**Figure 3:**
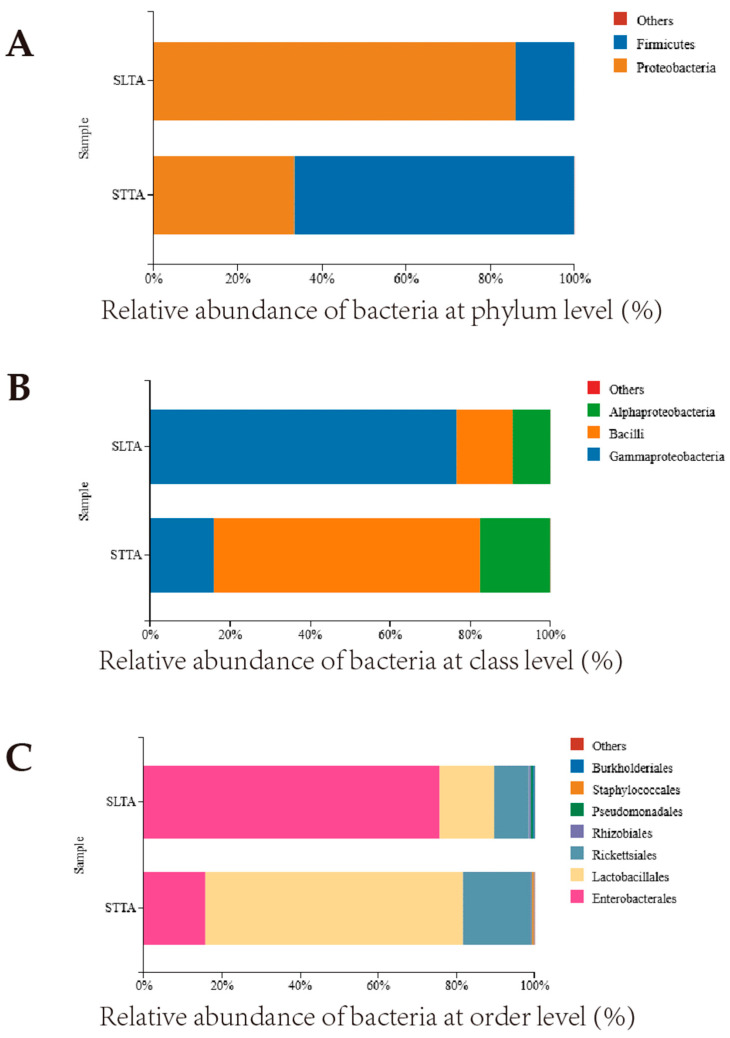

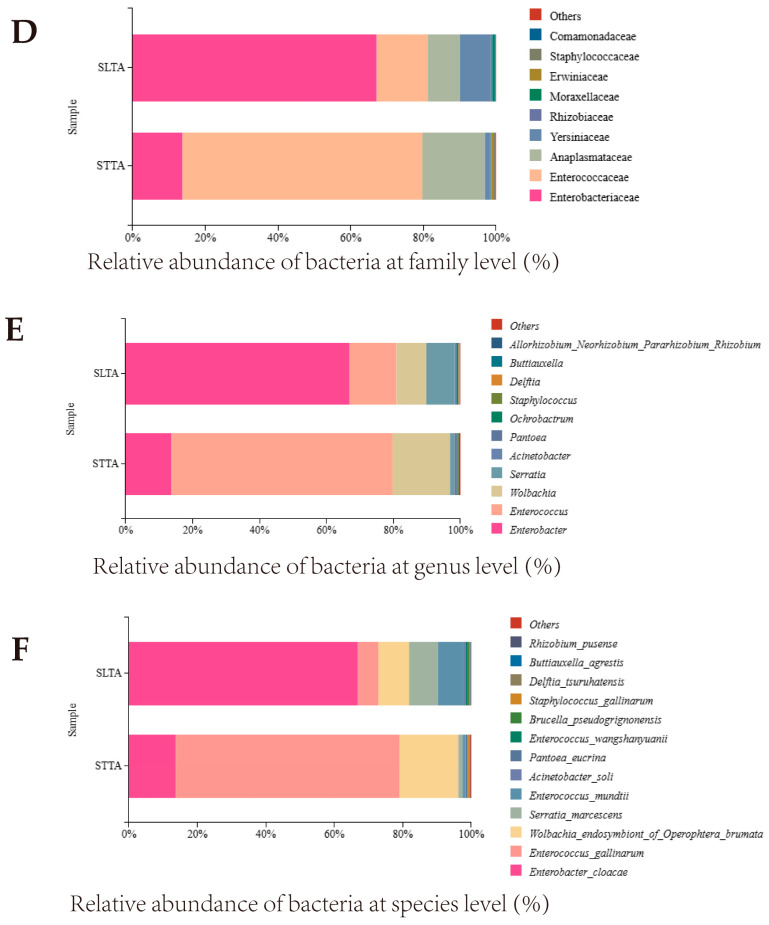
Analysis of different classification levels of gut flora of SLTA and STTA (SLTA, *T. absoluta* of tomato strain; STTA, *T. absoluta* of potato strain), (**A**) Phylum level; (**B**) Class level; (**C**) Order level; (**D**) Family level; (**E**) Genus level; (**F**) Species level.

Based on the SILVA ribosomal RNA database, the composition of the gut bacteria of *T. absoluta* fed on two host plants was analyzed at the taxonomic level (Figure 3). The SLTA was annotated to 4 phyla, 5 classes, 15 orders, 23 families, 31 genera, and 43 species; the STTA was annotated to 5 phyla, 6 classes, 16 orders, 29 families, 38 genera, and 52 species. At the phylum level (Figure 3A), the dominant phylum of SLTA and STTA were different: SLTA was Proteobacteria (85.9%), STTA was Firmicutes (66.5%); At the class level (Figure 3B), the dominant class of SLTA was Gammaproteobacteria (76.5%) and STTA was Bacilli (66.5%); At the order level the two’s dominant were also different (Figure 3C), the dominant order of SLTA was Enterobacterales (75.6%) and STTA was Lactobacillales (66.0%); SLTA and STTA have different dominant families at the family level (Figure 3D): SLTA is Enterobacteriaceae (67.1%) and STTA is Enterococcaceae (65.9%); The dominant bacteria of SLTA and STTA were also different at the genus level (Figure 3E), SLTA is *Enterobacter* (66.9%), STTA is *Enterococcus* (65.9%). At the species level (Figure 3F), *Enterobacter cloacae* (66.9%) is mainly enriched in SLTA. However, *Enterococcus gallinarum* (65.3%) is mainly distributed in STTA.

**Figure 4:**
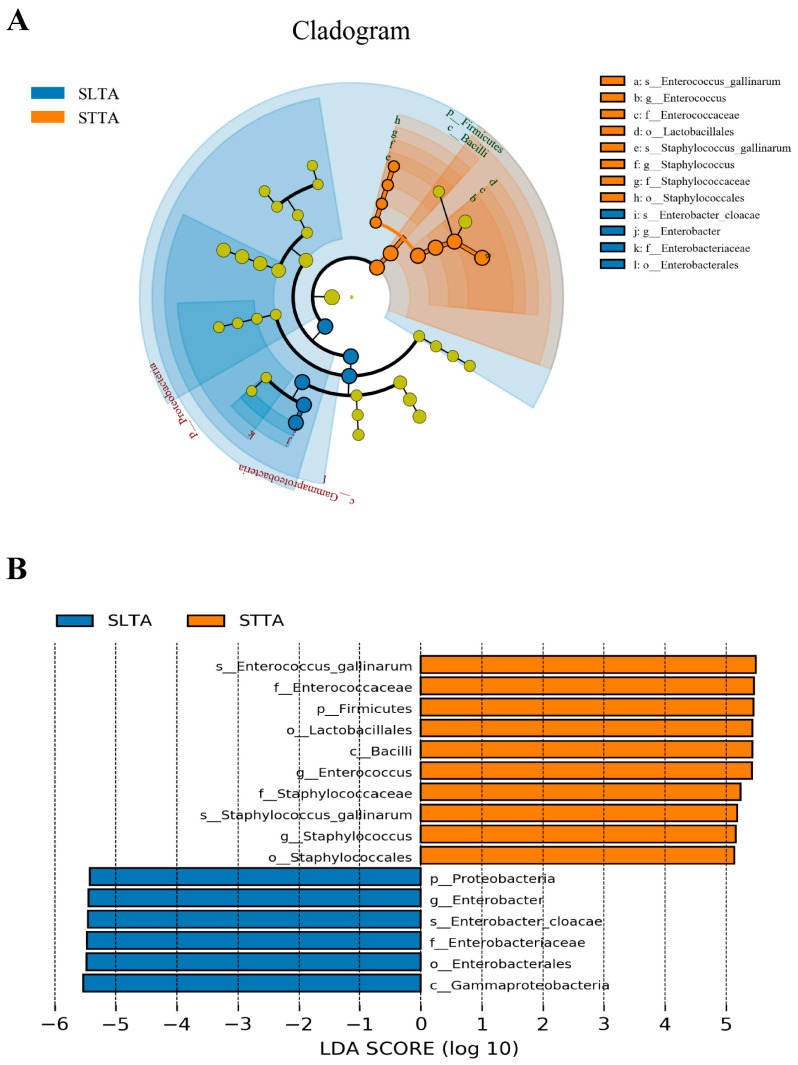
Based on the analysis of LEfSe, the difference in gut bacterial biomarkers of SLTA and STTA was analyzed (SLTA, *T. absoluta* of tomato strain; STTA, *T. absoluta* of potato strain). (**A**) Cladogram of bacterial biomarkers, the circle from the innermost ring (Phylum level) to the outermost ring (Strain level), in the (**A**), circles from center to outward layers represent taxonomic level from phylum to species. The node on circles represents a term on corresponding taxonomic level. The size of the dots indicates relative abundance. Colouring: small yellow nodes indicate bacterial taxa that were not significantly different in guts of larvae fed different hosts; Otherwise, the nodes were coloured according to the group with the highest relative abundance (blue was SLTA; orange was STTA), which helps visualize the relevance of different biological aspects; (**B**) Bacterial taxa with LDA score analysis (LDA > 4 and *p* < 0.05) in SLTA and STTA.

LEfSe was used to analyze the differential bacteria in the gut tract of *T. absoluta* treated with different hosts; the difference in the abundance of biomarker bacteria can represent the effect of exposure to different host plants on the gut bacteria of the *T. absoluta*. *E. cloacae* was the most abundant species in the tomato group, *E. gallinarum* and *S. gallinarum* were the most abundant species in the potato group (Figure 4A). The LDA score of *E. cloacae* in the tomato group was the highest and was greatly affected by the differences between the two groups, and it was identified as the bacterial biomarker. The LDA value of *E. gallinarum* was greater than that of other taxonomic units and had a greater impact on the differences between the two groups. In addition, *S. gallinarum* can serve as the second bacterial biomarker of the potato group (Figure 4B).

Kyoto Encyclopedia of Genes and Genomes (KEGG) based function prediction is a powerful analysis to reveal how metabolic functions are changed in a population for adaptation to the environment [47]. In order to analyze the function of bacterial biomarkers, KEGG level 1 analysis showed that the three types of bacteria were mainly related to metabolism (Figure S4A). KEGG level 2 was used to analyze the secondary metabolic pathways in which bacterial biomarkers were located; it was found that the three bacteria had high enrichment in the four metabolic pathways of carbohydrate metabolism, amino acid metabolism, membrane transport, and energy metabolism (Figure S4B). KEGG pathway level 3 analysis of the carbohydrate metabolism pathway with the highest relative abundance enriched by the main KEGG pathway level 2 showed that *E. cloacae* are mainly related to amino sugar and nucleotide sugar metabolism, pyruvate metabolism, starch and sucrose metabolism; *E. gallinarum* is mainly enriched in pyruvate metabolism and glycolysis/gluconeogenesis pathways; *S. gallinarum* mainly exists on amino sugar and nucleotide sugar metabolism, fructose and mannose metabolism and glycolysis/gluconeogenesis pathways (Figure S4C). The KEGG pathway enrichment analysis showed that the bacterial biomarkers mainly affected the adaptability of *T. absoluta* to host plants by affecting *T. absoluta*’s sugar metabolism.

### 3.3. Analysis of the Composition and Structure of Culturable Bacteria in the Gut of T. absoluta Larvae

**Figure 5:**
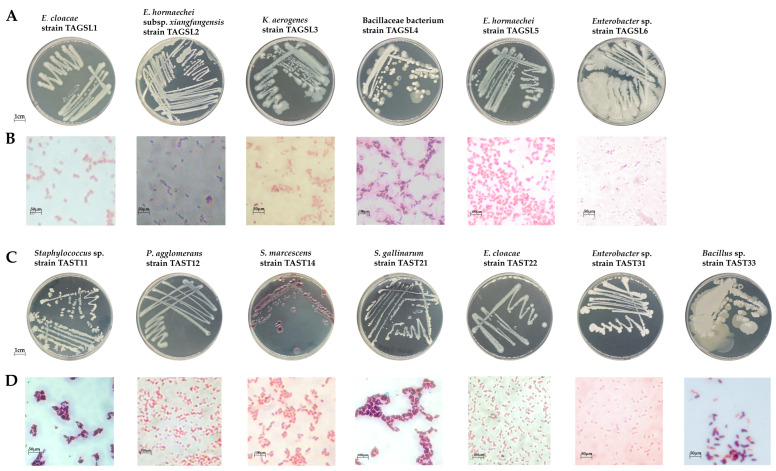
Colony morphology and cell morphology of some bacteria in *SLTA and STTA* gut: (**A**,**C**) Colony morphology; (**B**,**D**) Cell morphology; (SLTA, *T. absoluta* of tomato strain; STTA, *T. absoluta* of potato strain).

Furthermore, to confirm and detect the bacteria composition from SMRT, we isolated the bacteria from a gut tract of *T. absoluta* larvae fed with two different hosts. In total, 6 strains of bacteria were isolated from larvae fed with tomato; one strain of Gram-positive bacteria was long rod-shaped, and five strains of Gram-negative bacteria were short rod-shaped (Figure 5A,B, Table S2). Besides, 7 strains of bacteria were isolated from the gut of *T. absoluta* larvae in the potato group. Three strains of Gram-positive bacteria were spherical, and four strains of Gram-negative bacteria were found by Gram staining. Among them, one cell was spherical rod-shaped, and four were short rod-shaped (Figure 5C,D, Table S2).

Molecular identification of bacteria isolated and cultured from the gut of *T. absoluta*: 6 strains of bacteria isolated from the gut tract of SLTA were analyzed by sequencing and comparison, which belonged to 2 phyla, 2 families, 3 genera, and 6 species. Among them, Firmicutes has 1 family and 1 genus, namely *Bacillus* of Bacillaceae, and Proteobacteria has 1 family and 2 genera, including *Enterobacter* and *Klebsiella* of Enterobacteriaceae (Table 1). The 7 strains of bacteria isolated from the gut of STTA belonged to 2 phyla, 5 families, 5 genera, and 7 species. Among them, there are 2 families and 2 genera of Firmicutes, which belong to *Bacillus* and *Staphylococcus* of Staphylococcaceae. There are 3 families and 3 genera of Proteobacteria, which are *Enterobacter* of Enterobacteriaceae, *Pantoea* of Erwiniaceae, and *Serratia* of Yersiniaceae (Table 1). The bacteria identified in the gut tract of SLTA and STTA by traditional culture method were all consistent with the bacterial composition analysis in SMRT analysis. Among them, *Enterobacter cloaca* identified in the SLTA group was consistent with the dominant bacteria of SLTA in SMRT analysis (Figure 3F) and the bacterial biomarkers (Figure 4). *Staphylococcus gallinis* identified in the STTA group were consistent with the STTA bacterial biomarker in the SMRT analysis. However, the dominant and bacterial biomarker *E. gallinarum* of STTA was not isolated and cultured in vitro (Figure 5).

The phylogenetic evolutionary tree indicates that the gut-culturable bacteria in the tomato group were divided into two branches. The first branch consisted of Enterobacteriaceae bacteria from Proteobacteria, and the other clade consisted of bacteria from the family Bacillus of Firmicutes (Figure S5A). The enteric culturable bacteria in the potato group were divided into two branches. One branch consisted of Enterobacteriaceae, Erwinobacteriaceae, and Yersinobacteriaceae of Proteobacteria, while the second clade consisted of bacteria from the family Staphylococcaceae and the family Bacillus of Firmicutes (Figure S5B).

### 3.4. Gut Bacterial Biomarkers Improve the Feeding Ability of T. absoluta

**Figure 6:**
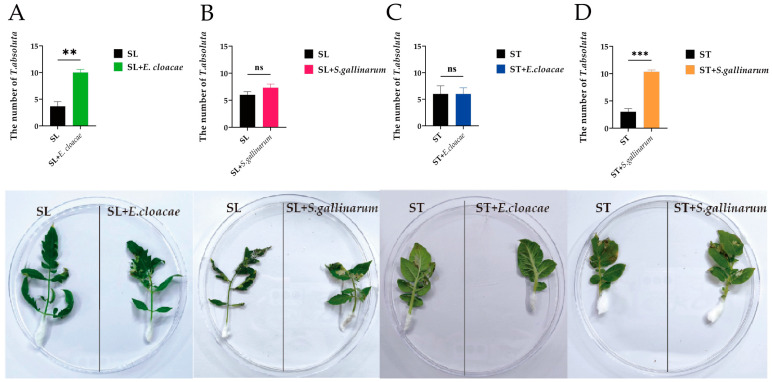
Analysis of feeding selection of *T. absoluta* of tomato strain (SLTAs) and *T. absoluta* of potato strain (STTAs) on leaves of different bacteria treatments; (**A**) SLTAs’ feeding selection analysis of tomato leaves treated with *E. cloacae*; (**B**) SLTAs’ feeding selection analysis of tomato leaves treated with *S. gallinarum*; (**C**) STTAs’ feeding selection analysis of potato leaves treated with *E. cloacae*; (**D**) STTAs’ feeding selection analysis of potato leaves treated with *S. gallinarum.* Data analysis was performed using an independent sample *t*-test, “ns” represents no difference, “**” represents *p* < 0.01; “***” represents *p* < 0.001.

Compared with the SL, SLTAs significantly tended to SL + *E. cloacae* (*Enterobacter cloacae* strain TAGSL1, PQ044797) (*p* = 0.004; Figure 6A), while SL + *S. gallinarum* (*Staphylococcus gallinarum* strain TAST21, PQ045250) had no significant difference compared with SL (*p* = 0.205; Figure 6B); At the same time, there was no significant difference between STTAs feeding ST + *E. cloacae* and ST (*p* = 1.000; Figure 6C), but STTAs tended to feed ST + *S. gallinarum* more than the ST (*p* < 0.001; Figure 6D). This shows that after long-term artificial domestication, the bacterial biomarkers of different strains of *T. absoluta* are beneficial in improving the adaptability to the original plant.

## 4. Discussion

Plants are related to life activities such as insect host selection, individual development, survival, and oviposition [48]. *T. absoluta* has a wide range of hosts, up to 11 families, and 50 species of plants [37], mainly including tomato, potato, eggplant, pepper, nightshade, and other Solanaceae plants [37]. Many studies have suggested that *T. absoluta* has a high preference for tomatoes. *T. absoluta* is more inclined to lay eggs and choose to hatch larvae on tomato leaves, and the early development time of *T. absoluta* adult on tomato is significantly shorter than that of eggplant [49]. The growth index of eggs, larvae, pupae, and total immature stage (egg-adult emergence) of *T. absoluta* was the highest on tomato leaves, followed by tomato fruits, potato leaves, eggplant leaves, etc. [50]. The results of the oviposition selection study of *T. absoluta* showed that tomato was the most suitable host, followed by potato, eggplant, and nightshade, and *T. absoluta* the highest oviposition was tomato, followed by nightshade, bell pepper, and capsicum, and the survival rate was higher on tomato [3,51]. In this study, the larval duration of *T. absoluta* fed on tomatoes was shorter than that fed on potatoes, while the adult duration was the opposite. The number of eggs laid by a single female of *T. absoluta* feeding on tomatoes was significantly higher than that of the potato group. This phenomenon indicated that tomatoes could assist in the growth and development of *T. absoluta* in a short time compared with potatoes. Prolonging the oviposition time was conducive to population expansion; that is, tomato was the most suitable host for *T. absoluta*; this is consistent with the above results.

Plant tissues contain a number of secondary metabolites and toxic defense substances, which are often not conducive to the normal growth and development of insects [52]. In order to adapt to different host plants, herbivores have evolved a series of strategies to overcome plant defenses, including changes in gut bacteria, which can promote the adaptability and metabolic function of different host plants [53,54]. Lü et al. [55] found that the gut tract of *Henosepilachna vigintioctopunctata* larvae fed on eggplant (*Solanum melongena*) was mainly enriched in *Lactococcus,* while the bacterial abundance of *Ochrobactrum* in the gut tract of *Solanum nigrum* was the highest. Lateef et al. [56] used LEfSe analysis and showed that the differential bacteria in the larvae were *Enterococcus*, *Enterobacter*, *Lactococcus*, *Klebsiella,* and *Wiessella* compared with the distribution of the adult flora of the *T. absoluta*. Wang et al. [57] analyzed the flora of *T. absoluta* in three geographic populations using 16S rRNA and showed that the main phylums were Firmicutes and Proteobacteria. The bacteria isolated and identified by Eski et al. [58] with resistance to *T. absoluta* larvae were mainly Firmicutes and Proteobacteria. At the phylum classification level, Wang et al. and Eski et al. [57,58] are consistent with our results. In this research, the dominant bacteria and bacterial biomarkers in the gut of *T. absoluta* feeding on tomatoes were both *E. cloacae*, while the dominant bacteria in the gut tract of *T. absoluta* fed on potatoes was *E. gallinarum*, and the bacterial biomarkers of *E. gallinarum* and *S. gallinarum*. There were also significant differences in the structure and abundance of gut bacteria after *T. absoluta* fed on two plants of Solanaceae, tomato and potato.

Insects obtain and preserve bacteria from the external environment or hosts and use them to improve their ability to adapt to the external environment or host plants [59,60]. For example, the gut bacteria *E. cloacae* contributed to the growth and development of *S. frugiperda* in harsh dietary environments [61] and also enhanced the sensitivity of the oriental armyworm, *M. separata*, to Bt toxins in genetically modified maize [62]. Therefore, *E. cloacae* plays an essential role in the adaptation of insects to host plants. Particularly, *T. absoluta*’s host is solanaceae, which contains a variety of natural toxins such as solanine, alkaloids, flavonoids, and nicotine [63]. At the same time, in our research, *E. cloacae* could effectively improve the adaptability of the tomato stain of *T. absoluta* to tomato (Figure 6A). According to the report, Enterococcus bacteria can protect the host from external pathogens and non-symbiotic microorganisms and improve tolerance to toxic foods [64,65]. For instance, two Enterococcus bacteria in the gut of *S. frugiperda* contribute to the detoxification of host plants by *S. frugiperda* [66]. This may be related to the ability of these Enterococcus bacteria to improve the resistance, tolerance, and detoxification of *T. absoluta*. While most bacteria show high abundance as biomarkers for specific dietary characteristics of insects, some are considered core taxa [67]. Moreover, some low-abundance bacterial genera were also found to be potential biomarkers, suggesting that both high-abundance and low-abundance bacterial groups play an important role in shaping the dietary patterns of insects [68]. *S. gallinarum* in the gut microbiota has been shown to improve the host adaptability of *Callosobruchus maculatus* by encoding many digestive enzymes that degrade the secondary metabolites of toxic plants [69]. *S. gallinarum* KX912244 in the gut tract of silkworms can produce Staphyloxanthin with a strong antioxidant function to improve the adaptability of host insects [70]. *Staphylococcus*, which has protease activity in *Anticarsia gemmatalis*, can assist insects in degrading the virulence of plant trypsin inhibitors to overcome plant defense [71]. In this study, during our in vitro isolation, we identified *E. cloacae* and *S. gallinarum* but not *E. gallinarum*. We found that the biomarker gut bacteria *S. gallinarum* of STTA is helpful in improving the adaptability of STTA to potatoes (Figure 6D). This may be related to the fact that the *S. gallinarum* can promote the synthesis of detoxification metabolic enzymes and degrade plant defense substances; this is also consistent with the results of KEGG analysis that these two bacteria are mainly related to metabolism (Figure S4). Gohl et al. [31] and Xin et al. [32] studies have shown that the gut flora of insects fed on host plants is different from that of artificial diets. Feeding *T. absoluta* with non-host plants as a control can highlight the effect of plants on gut bacteria. However, the oviposition and hatching of *T. absoluta* cannot be replaced by non-host plants. It is urgent to develop non-host plants or artificial diets for the construction of sterile strains to assist in the verification of the function of single bacteria.

In summary, based on the differences in the growth and development of *T. absoluta* affected by different host plants, this paper explored the composition, abundance changes, bacterial biomarkers types, and functions of gut bacteria of *T. absoluta* after feeding on different host plants and analyzed the function of gut bacterial biomarkers to infer the role of insect gut bacteria in insect growth and development and adaptation to host plants. Therefore, this study provides strain resources for the development and utilization of functional bacteria, contributes to deep analysis of the mechanism of gut bacteria in mediating insect-plant interaction, and also provides new ideas for the green and effective biological control of *T. absoluta*.

## Figures and Tables

**Table 1:** Results of comparison of culturable bacterial strains from the gut tract of SLTA and STTA with typical strains in the database.

Host Plant	Strain No.	Accession No.	Phylum	Family	The Most Similar Strain	Identity/%
*Solanum lycopersicum*	*Bacillaceae bacterium* strain TAGSL4	PQ044800	Firmicutes	Bacillaceae	*Bacillus* sp. DB89 (2010) (HM566969.1)	98
	*Enterobacter cloacae* strain TAGSL1	PQ044797	Proteobacteria	Enterobacteriaceae	*E. cloacae* strain 175-d blue (MN208123.1)	98
	*Enterobacter hormaechei* subsp. *xiangfangensis* strain TAGSL2	PQ044798			*E. hormaechei* subsp. *xiangfangensis* strain HB08 (PP838251.1)	99
	*Klebsiella aerogenes* strain TAGSL3	PQ044799			*K. aerogenes* strain DAS50 (MH819723.1)	98
	*Enterobacter hormaechei* strain TAGSL5	PQ044801			*E. hormaechei* strain LZH-G4 (OL687492.1)	99
	*Enterobacter* sp. strain TAGSL6	PQ044802			*Enterobacter* sp. C49 (EU563349.1)	99
*Solanum tuberosum*	*Staphylococcus* sp. strain TAST11	PQ045247	Firmicutes	Staphylococcaceae	*Staphylococcus* sp. strain PE4 (OP108436.1)	99
	*Staphylococcus gallinarum* strain TAST21	PQ045250			*S. gallinarum* strain OOM34 (MH542297.1)	99
	*Bacillus* sp. strain TAST33	PQ045253		Bacillaceae	*Bacillus* sp. strain ESA 675 (MT482581.1)	97
	*Pantoea agglomerans* strain TAST12	PQ045248	Proteobacteria	Erwiniaceae	*P. agglomerans* strain T224 (KC764985.1)	99
	*Serratia marcescens* strain TAST14	PQ045249		Yersiniaceae	*S. marcescens* strain Atecer7A (MT386168.1)	99
	*Enterobacter cloacae* strain TAST22	PQ045251		Enterobacteriaceae	*E. cloacae* strain T137 (KY285207.1)	94
	*Enterobacter* sp. strain TAST31	PQ045252			*Enterobacter* sp. RITF1201 (KF445140.1)	97

## Data Availability

The *16S rRNA* genes sequence accession number of gut bacteria in vitro culture of 6 tomato strains of *T. absoluta* was PQ044797-PQ044802; The *16S rRNA* genes sequence accession number of gut bacteria in vitro culture of 7 potato strains of *T. absoluta* was PQ045247-PQ045253; The raw data of the SMRT sequencing of *T. absoluta’s* gut bacteria was uploaded to NCBI Sequence Read Archive (SRA) database with accession number PRJNA1137249.

## Supplementary Materials

The following supporting information can be downloaded at https://www.mdpi.com/article/10.3390/insects15100795/s1: Figure S1. Venn diagram of the otu division of intestinal bacteria for SLTA and STTA. Figure S2. Analysis of β diversity index of gut bacteria of SLTA and STTA. Figure S3. Analysis of UPGMA and abundance at different classification levels of gut bacteria of *T. absoluta* treated with two host plants. Figure S4. KEGG pathway analysis of bacterial biomarkers on different levels.. Figure S5. Phylogenetic tree based on gut bacteria of SLTA and STTA. Table S1. Statistics on sequencing data of gut samples of SLTA and STTA. Table S2. Colony morphology and cultural characteristics of bacteria in SLTA and STTA.

## References

[1] Chatterjee D., Lesko T., Peiffer M., Elango D., Beuzelin J., Felton G., Chopra S. (2023). Sorghum and maize flavonoids are detrimental to growth and survival of fall armyworm *Spodoptera frugiperda*. J. Pest Sci..

[2] Zhang M., Ren X.L., Hu H.Y., Wang D., Song X.P., Ma Y., M X.Y. (2023). Evaluation of cotton, sweet potato, peanut, and black nightshade on the fitness of *Spodoptera litura* Fabricius (Lepidoptera: Noctuidae). Int. J. Trop. Insect Sci..

[3] Idriss G.E.A., Plessis H.D., Khamis F.M., Ekesi S., Tanga C.M., Mohamed S.A. (2020). Host range and effects of plant species on preference and fitness of *Tuta absoluta* (Lepidoptera: Gelechiidae). J. Econ. Entomol..

[4] Pan Q.J., Shikano I., Liu T.X., Felton G.W. (2023). *Helicoverpa zea*–associated gut bacteria as drivers in shaping plant anti-herbivore defense in tomato. Microb. Ecol..

[5] Acevedo F.E., Peiffer M., Tan C.W., Stanley B.A., Stanley A., Wang J., Jones A.G., Hoover K., Rosa C., Luthe D. (2017). Fall armyworm-associated gut bacteria modulate plant defense responses. Mol. Plant Microbe. In..

[6] Gershenzon J., Ullah C. (2022). Plants protect themselves from herbivores by optimizing the distribution of chemical defenses. Proc. Natl. Acad. Sci. USA.

[7] Liu H.P., Yang Q.Y., Liu J.X., Haq I.U., Li Y., Zhang Q.Y., Attia K.A., Abushady A.M., Liu C.Z., Lv N. (2023). Host plant-mediated effects on *Buchnera* symbiont: Implications for biological characteristics and nutritional metabolism of pea aphids (*Acyrthosiphon pisum*). Front. Plant Sci..

[8] Sun S.L., Yang Z., Ren J.C., Liu T.X., Jing X.F. (2023). Fitness of Nutrition Regulation in a Caterpillar Pest *Mythimna separata* (Walker): Insights from the Geometric Framework. Insects.

[9] Abendroth J.A., Moural T.W., Wei H., Zhu F. (2023). Roles of insect odorant binding proteins in communication and xenobiotic adaptation. Front. Insect Sci..

[10] Picimbon J.F. (2020). Interpopulational variations of odorant-binding protein expression in the black cutworm moth, *Agrotis ipsilon*. Insects.

[11] Conchou L., Anderson P., Birgersson G. (2017). Host plant species differentiation in a polyphagous moth: Olfaction is enough. J. Chem. Ecol..

[12] Hafeez M., Li X.W., Chen L.M., Ullah F., Huang J., Zhang Z.J., Zhang J.M., Siddiqui J.A., Zhou S.X., Ren X.Y. (2023). Molecular characterization and functional analysis of cytochrome P450-mediated detoxification *CYP302A1* gene involved in host plant adaptation in *Spodoptera frugieprda*. Front. Plant Sci..

[13] He L., Shi Y., Ding W.B., Huang H., He H.L., Xue J., Gao Q., Zhang Z.X., Li Y.Z., Qiu L. (2023). Cytochrome P450s genes *CYP321A9* and *CYP9A58* contribute to host plant adaptation in the fall armyworm *Spodoptera frugiperda*. Pest Manag. Sci..

[14] Zhi J.R., Liu L., Hou X.L., Xie W., Yue W.B., Zeng G. (2021). Role of digestive enzymes in the adaptation of *Frankliniella occidentalis* to preferred and less-preferred host plants. Entomol. Exp. Appl..

[15] Zhang A.Y., Li T., Yuan L.S., Tan M.T., Jiang D., Yan S.C. (2023). Digestive characteristics of *Hyphantria cunea* larvae on different host plants. Insects.

[16] Zhang S.K., Song F., Wang J., Li X.Y., Zhang Y.X., Zhou W.W., Xu L.T. (2024). Gut microbiota facilitate adaptation of invasive moths to new host plants. ISME J..

[17] Santos-Garcia D., Mestre-Rincon N., Zchori-Fein E., Morin S. (2020). Inside out: Microbiota dynamics during host-plant adaptation of whiteflies. ISME J..

[18] Xia X.F., Wang Q., Gurr G.M., Vasseur L., Han S.C., You M.S. (2023). Gut bacteria mediated adaptation of diamondback moth, *Plutella xylostella*, to secondary metabolites of host plants. mSystems.

[19] Guo J., Wu J., Deng X.Y., Lin L.B., Liu S., Li J.L. (2015). Advances in functional studies of insect intestinal flora. Chin. Bull. Entomol..

[20] Raza M.F., Wang Y.C., Cai Z.H., Bai S., Yao Z.C., Awan U.A., Zhang Z.Y., Zheng W.W., Zhang H.Y. (2020). Gut microbiota promotes host resistance to low-temperature stress by stimulating its arginine and proline metabolism pathway in adult *Bactrocera dorsalis*. PLoS Pathog..

[21] Muñoz-Benavent M., Pérez-Cobas A.E., García-Ferris C., Moya A., Latorre A. (2021). Insects’ potential: Understanding the functional role of their gut microbiome. J. Pharm. Biomed. Anal..

[22] Tafesh-Edwards G., Eleftherianos I. (2023). The role of *Drosophila* microbiota in gut homeostasis and immunity. Gut Microbes.

[23] Zhang Q., Wang S., Zhang X., Zhang K.X., Liu W.J., Zhang R.L., Zhang Z. (2021). *Enterobacter hormaechei* in the intestines of housefly larvae promotes host growth by inhibiting harmful intestinal bacteria. Parasite Vector.

[24] Franks K., Kooienga E., Sanders M., Pendarvis K., Yang F., Tomberlin J.K., Jordan H.R. (2021). The effect of *Rhodococcus rhodochrous* supplementation on black soldier fly (Diptera: Stratiomyidae) development, nutrition, and waste conversion. J. Insects Food Feed.

[25] Wang W.X., Zhu Y.H., Lai F.X., Wei Q., Wan P.J., He J.C., Fu Q. (2023). Effects of introduction of *Bacillus* spp. on the microbiota and growth and development of the brown planthopper, *Nilaparvata lugens* (Hemiptera: Delphacidae). Acta. Entomol. Sin..

[26] Shi Z.H., Hou Y.M. (2020). Research progress on the mechanism of interaction between insects and their intestinal flora and its application prospects in pest management. J. Enviro. Entomol..

[27] Härer A., Mauro A.A., Laurentino T.G., Rosenblum E.B., Rennison D.J. (2023). Gut microbiota parallelism and divergence associated with colonisation of novel habitats. Mol. Ecol..

[28] Li Q., Fan J., Sun J.X., Wang M.Q., Chen J.L. (2018). Plant-mediated horizontal transmission of *Hamiltonella defensa* in the wheat aphid *Sitobion miscanthi*. J. Agric. Food Chem..

[29] Pons I., Renoz F., Noël C., Hance T. (2019). Circulation of the cultivable symbiont *Serratia symbiotica* in aphids is mediated by plants. Front. Microbiol..

[30] Zhang Y.X., Zhang S.K., Xu L.T. (2023). The pivotal roles of gut microbiota in insect plant interactions for sustainable pest management. npj Biofilms Microbi..

[31] Gohl P., LeMoine C.M.R., Cassone B.J. (2022). Diet and ontogeny drastically alter the larval microbiome of the invertebrate model *Galleria mellonella*. Can. J. Microbiol..

[32] Xin L., Chen Y., Rong W., Qin Y., Li X., Guan D. (2024). Gut microbiota analysis in silkworms (*Bombyx mori*) provides insights into identifying key bacterials for inclusion in artificial diet formulations. Anim. Basel.

[33] Deng J.D., Wang H.C., Xu W.K., Xu L.T., Tang Y.P., Zhang L.W. (2022). Effects of different host plants on intestinal bacterial communities of *Hyphantria cunea*. J. Plant Prot..

[34] Yuan X.Q., Zhang X., Liu X.Y., Dong Y.L., Yan Z.Z., Lv D.B., Wang P., Li Y.P. (2021). Comparison of gut bacterial communities of *Grapholita molesta* (Lepidoptera: Tortricidae) reared on different host plants. Int. J. Mol. Sci..

[35] Lv D.B., Liu X.Y., Dong Y.L., Yan Z.Z., Zhang X., Wang P., Yuan X.Q., Li Y.P. (2021). Comparison of gut bacterial communities of Fall Armyworm (*Spodoptera frugiperda*) reared on different host plants. Int. J. Mol. Sci..

[36] Rwomushana I., Pratt C., González-Moreno P., Beale T., Godwin J., Makale F., Day R., Kansiime M.K., Idah M., Murphy S.T. (2019). Tomato leaf miner (*Tuta absoluta*): Impacts and coping strategies for Africa. CABI Working Pap..

[37] Zhang G.F., Xian X.Q., Zhang Y.B., Zhang R., Ma D.Y., Liu W.X., Gao Y.H., Wang J., Yang Z.L., Li Q.H. (2020). Warning of the dispersal of a newly invaded alien species, tomato leaf miner *Tuta absoluta* (Meyrick), in China. Plant Prot..

[38] Mosher J.J., Bowman B., Bernberg E.L., Shevchenko O., Kan J.J., Korlach J., Kaplan L.A. (2014). Improved performance of the PacBio SMRT technology for 16S rDNA sequencing. J. Microbiol. Meth..

[39] Wagner J., Coupland P., Browne H.P., Lawley T.D., Francis S.C., Parkhill J. (2016). Evaluation of PacBio sequencing for full-length bacterial 16S rRNA gene classification. BMC Microbiol..

[40] Hou Q.C., Xu H.Y., Zheng Y., Xi X.X., Kwok L.Y., Sun Z.H., Zhang H.P., Zhang W.Y. (2015). Evaluation of bacterial contamination in raw milk, ultra-high temperature milk and infant formula using single molecule, real-time sequencing technology. J. Dairy Sci..

[41] Li W.C., Hou Q.C., Wang Y.J., Ma H.M., Liu Y.H., Zhao F.Y., Li J., Kwok L.Y., Yu J., Sun Z.H. (2018). Analysis of the gut microbial diversity of dairy cows during peak lactation by PacBio Single-Molecule Real-Time (SMRT) sequencing. Curr. Microbiol..

[42] Zhang Y.X., Fang J.K., Yang J.Y., Gao X.L., Dong L.Y., Zheng X., Sun L.G., Xia B., Zhao N., Ma Z.Y. (2022). Streptococcus mutans-associated bacteria in dental plaque of severe early childhood caries. J. Oral. Microbiol..

[43] Buchanan R.E., Gibbons N.E., Institute of Microbiology (1984). Bergey’s Manual of Determinative Bacteriology.

[44] Hu J.Y., Wu J.J., Wang X.Y., Huang J., Sun Q., Hu L.L., Yi Z.F., Chang Z.Y., Gao H.L., Niu Y.N. (2019). Isolation, identification and yield correlation of cellulose-producing strains. Microbiol. China.

[45] Liu J., Li N.W., Su S.H., Liu G.L., Gao S.J. (2023). Isolation, screening and identification of novel acetobacter for seedless wampee fruit vinegar fermentation. Food Sci. Technol..

[46] Yao Y.H., W W.Q., Du G.Z., Shen Y.F., Meng J.Z., Xiao W.X., Chen B. (2024). Structure, diversity and function prediction of intestinal tract bacterial composition of *Paralipsa gularis* (Zeller) a new maize pest. J. Southern Agric..

[47] Kanehisa M., Araki M., Goto S., Hattori M., Hirakawa M., Itoh M., Katayama T., Kawashima S., Okuda S., Tokimatsu T. (2007). KEGG for linking genomes to life and the environment. Nucleic Acids Res..

[48] Hu G.X., Gou W.S., Ma W.X., Kong J.H., Tang L., Sun Y.D. (2024). Effects of three host plants on growth, development, reproduction and longevity of *Leiometopon simyrides*. Plant Prot..

[49] Ingegno B.L., Candian V., Psomadelis I., Bodino N., Tavella L. (2017). The potential of host plants for biological control of *Tuta absoluta* by the predator *Dicyphus errans*. Bull. Entomol. Res..

[50] Negi S., Sharma P.L., Sharma K.C., Verma S.C. (2018). Effect of host plants on developmental and population parameters of invasive leaf miner, *Tuta absoluta* (Meyrick) (Lepidoptera: Gelechiidae). Phytoparasitica.

[51] Aparna S., Kumar A.R.V., Sotelo-cardona P., Srinivasan R. (2024). Host plant selection is linked to performance in *Phthorimaea absoluta* (Lepidoptera: Gelechiidae). Environ. Entomol..

[52] Erb M., Reymond P. (2019). Molecular interactions between plants and insect herbivores. Annu. Rev. Plant Biol..

[53] Yang Y.J., Liu X.G., Xu H.X., Liu Y.H., Lu Z.X. (2022). Effects of host plant and insect generation on shaping of the gut microbiota in the rice leaffolder, *Cnaphalocrocis medinalis*. Front. Microbiol..

[54] Benjamin G.H., Robert F.M., Richard T.L., Cécile P.F., John G.H., Noah K.W. (2015). Evolution of herbivory in Drosophilidae linked to loss of behaviors, antennal responses, odorant receptors, and ancestral diet. Proc. Natl. Acad. Sci. USA.

[55] Lü J., Guo W., Chen S.M., Guo M.J., Qiu B.L., Yang C.X. (2019). Host plants influence the composition of the gut bacteria in *Henosepilachna vigintioctopunctata*. PLoS ONE.

[56] Lateef A.A., Azeez A.A., Ren W., Hamisu H.S., Oke O.A., Asiegbu F.O. (2024). Bacterial biota associated with the invasive insect pest *Tuta absoluta* (Meyrick). Sci. Rep..

[57] Wang H., Xian X., Gu Y., Castañé C., Arnó J., Wu S., Wan F., Liu W., Zhang G., Zhang Y. (2022). Similar bacterial communities among different populations of a newly emerging invasive species, *Tuta absoluta* (Meyrick). Insects.

[58] Eski A., Erdoğan P., Demirbağ Z., Demir İ. (2024). Isolation and identification of bacteria from the invasive pest *Tuta absoluta* (Meyrick) (Lepidoptera: Gelechiidae) and evaluation of their biocontrol potential. Int. Microbiol..

[59] Yang Z.W., Luo J.Y., Men Y., Liu Z.H., Zheng Z.K., Wang Y.H., Xie Q. (2023). Different roles of host and habitat in determining the microbial communities of plant-feeding true bugs. Microbiome.

[60] Shan H.W., Xia X.J., Feng Y.L., Wu W., Li H.J., Sun Z.T., Li J.M., Chen J.P. (2024). The plant-sucking insect selects assembly of the gut microbiota from environment to enhance host reproduction. npj Biofilms Microbiomes.

[61] Wang P., He P.C., Fu W.D., Chu D. (2023). Adverse effects of high concentrations of two enteric bacteria on *Spodoptera frugiperda* and their benefits with respect to insect food quality. Entomol. Gen..

[62] Xu C., Luo J.Y., Wang L., Zhu X.Z., Xue H., Huang F.N.B., Gao X.K., Li D.Y., Zhang K.X., Chen R. (2023). Gut bacterial community and gene expression alterations induced by transgenic Bt maize contribute to insecticidal activity against *Mythimna separata*. J. Pest Sci..

[63] Liang K.H., Lu L.G., Zhu D.Z., Zhu H. (2017). Research progress of potato glucoside alkaloids. Food Res. Dev..

[64] Silverman N., Paquette N. (2008). The right resident bugs. Science.

[65] Vilanova C., Baixeras J., Latorre A., Porcar M. (2016). The generalist inside the specialist: Gut bacterial communities of two insect species feeding on toxic plants are dominated by *Enterococcus* sp.. Front. Microbiol..

[66] Gomes A.F.F., Almeida L.G., Cônsoli F.L. (2023). Comparative genomics of pesticide-degrading *Enterococcus* symbionts of *Spodoptera frugiperda* (Lepidoptera: Noctuidae) leads to the identification of two new species and the reappraisal of insect-associated *Enterococcus* species. Microb. Ecol..

[67] Zheng X., Zhu Q.D., Zhou Z.J., Wu F.T., Chen L.X., Cao Q.R., Shi F.M. (2021). Gut bacterial communities across 12 Ensifera (Orthoptera) at different feeding habits and its prediction for the insect with contrasting feeding habits. PLoS ONE.

[68] Benjamino J., Lincoln S., Srivastava R., Graf J. (2018). Low-abundant bacteria drive compositional changes in the gut microbiota after dietary alteration. Microbiome.

[69] Berasategui A., Moller A.G., Weiss B., Beck C.W., Bauchiero C., Read T.D., Gerardo N.M., Salem H. (2021). Symbiont genomic features and localization in the bean beetle *Callosobruchus maculatus*. Appl Environ. Microbiol..

[70] Barretto D.A., Vootla S.K. (2018). In vitro anticancer activity of Staphyloxanthin pigment extracted from *Staphylococcus gallinarum* KX912244, a gut microbe of Bombyx mori. Indian J. Microbiol..

[71] Visôtto L.E., Oliveira M.G.A., Ribon A.O.B., Mares-Guia T.R., Guedes R.N.C. (2009). Characterization and identification of proteolytic bacteria from the gut of the velvetbean caterpillar (Lepidoptera: Noctuidae). Environ. Entomol..

