# Supplementary material for: Role of Gut Bacteria in Enhancing Host Adaptation of Tuta absoluta to Different Host Plants
Source: Insects. 2024 Oct 13;15(10):795. doi: 10.3390/insects15100795 (PMC11508330; doi:10.3390/insects15100795)
Supplement: Supplementary file 1 [file insects-15-00795-s001.zip › insects-3171609-supplementary.pdf]

insects

Article

Adaptability analysis of gut bacteria from *Tuta absoluta* on different host plants

Supplementary Materials:

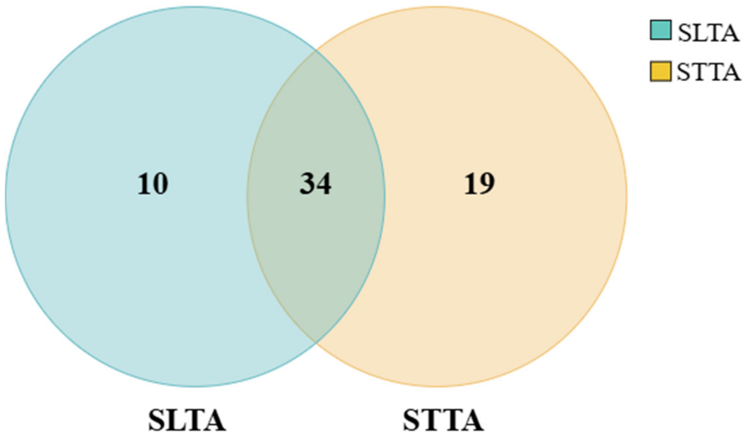

Figure S1. Venn diagram of the otu division of intestinal bacteria for SLTA and STTA. SLTA is *T. absoluta* fed on tomatoes; STTA is *T. absoluta* fed on potatoes.

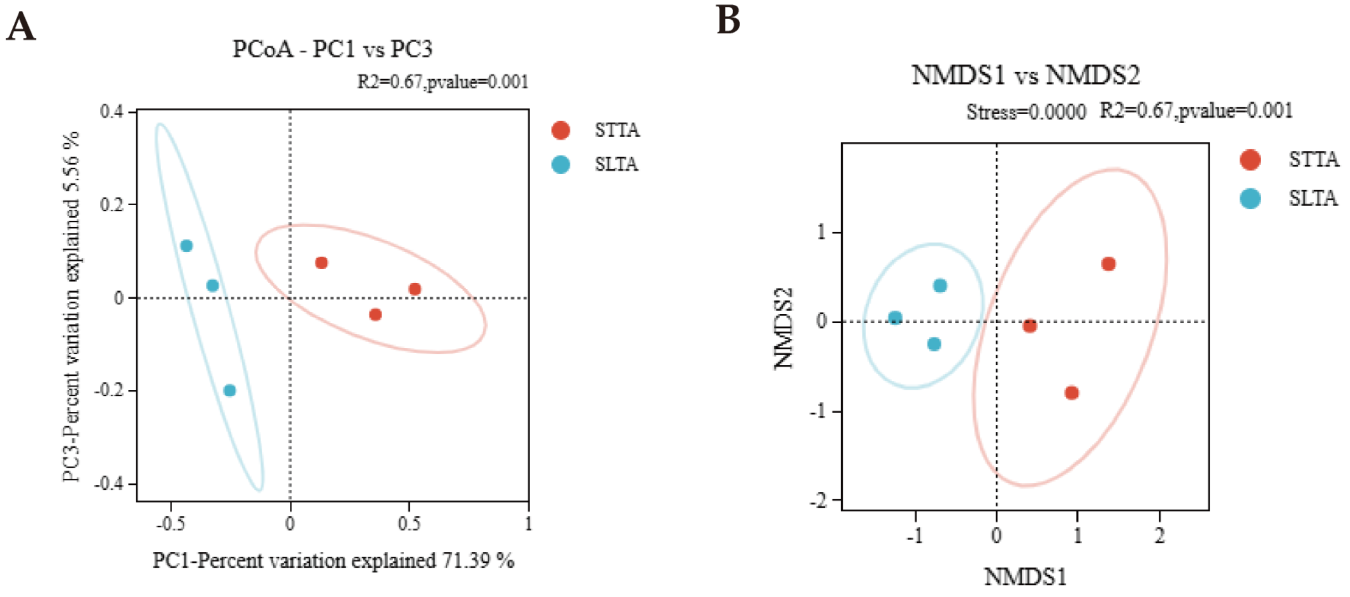

Figure S2. Analysis of β diversity index of gut bacteria of SLTA and STTA: (A) Principal coordinates analysis (PCoA), (B) Non-metric multi-dimensional scaling analysis (NMDS).

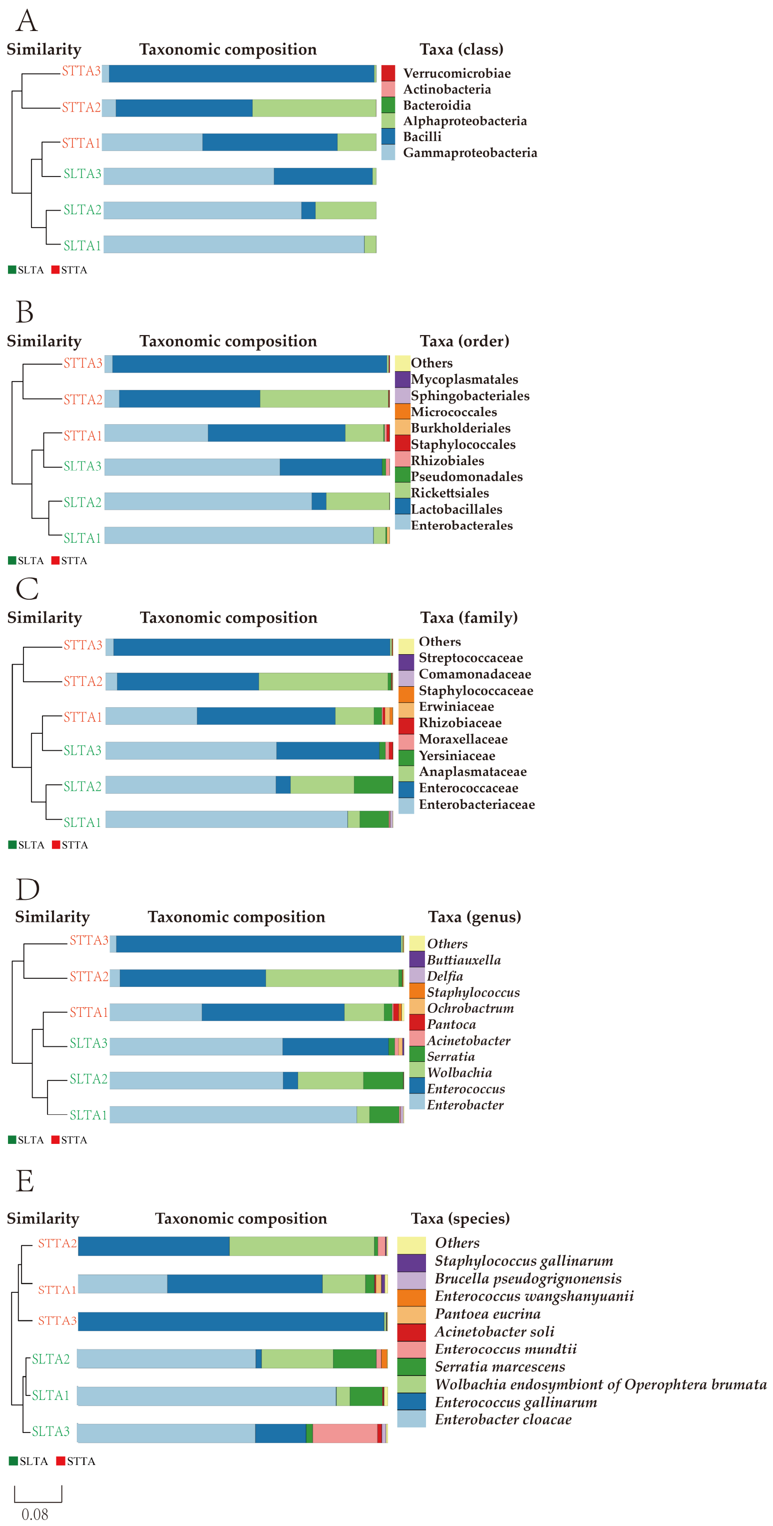

Figure S3. Analysis of UPGMA and abundance at different classification levels of gut bacteria of *T. absoluta* treated with two host plants.

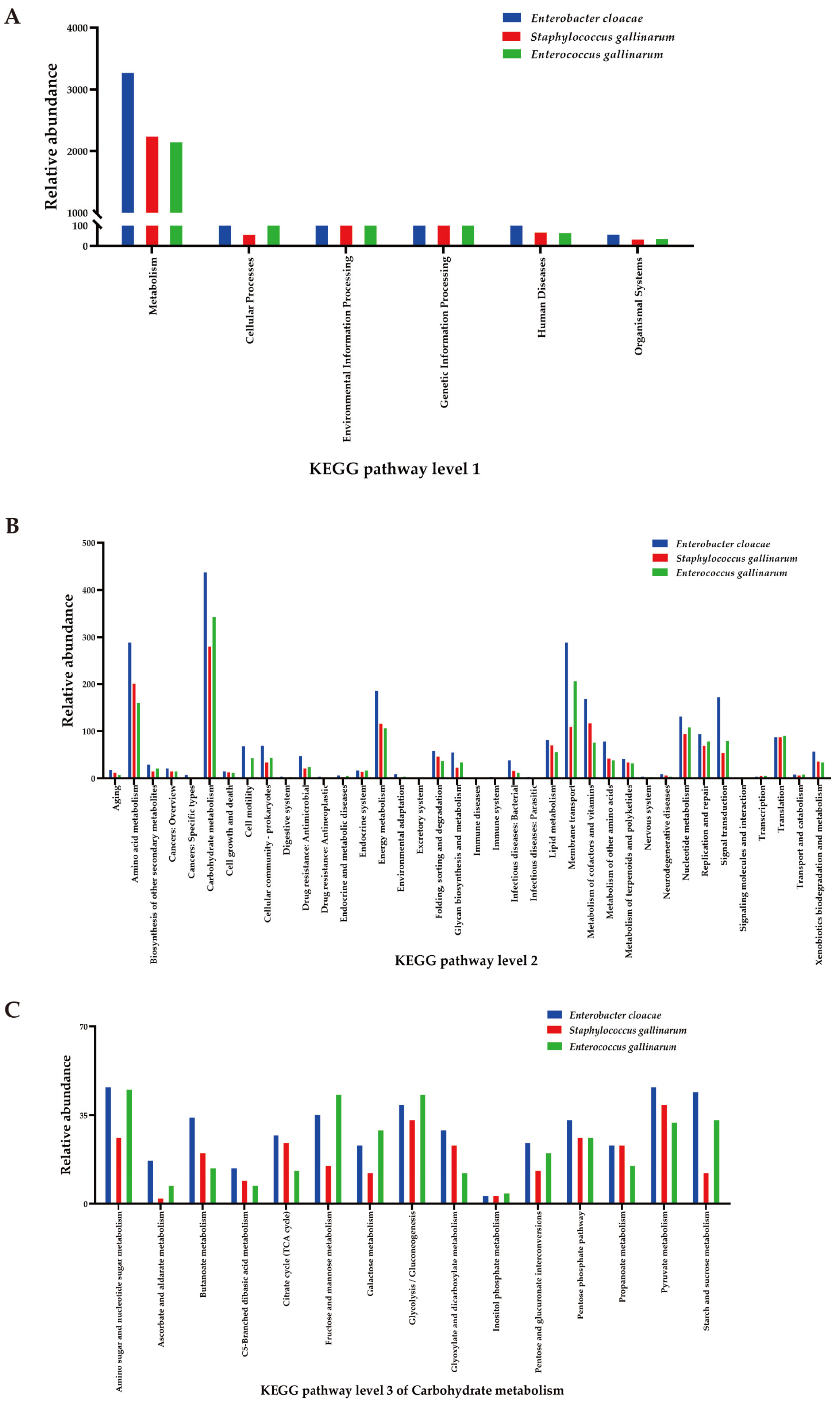

Figure S4. KEGG pathway analysis of bacterial biomarkers on different levels: (A) level 1; (B) level 2; (C) level 3 of carbohydrate metabolism.

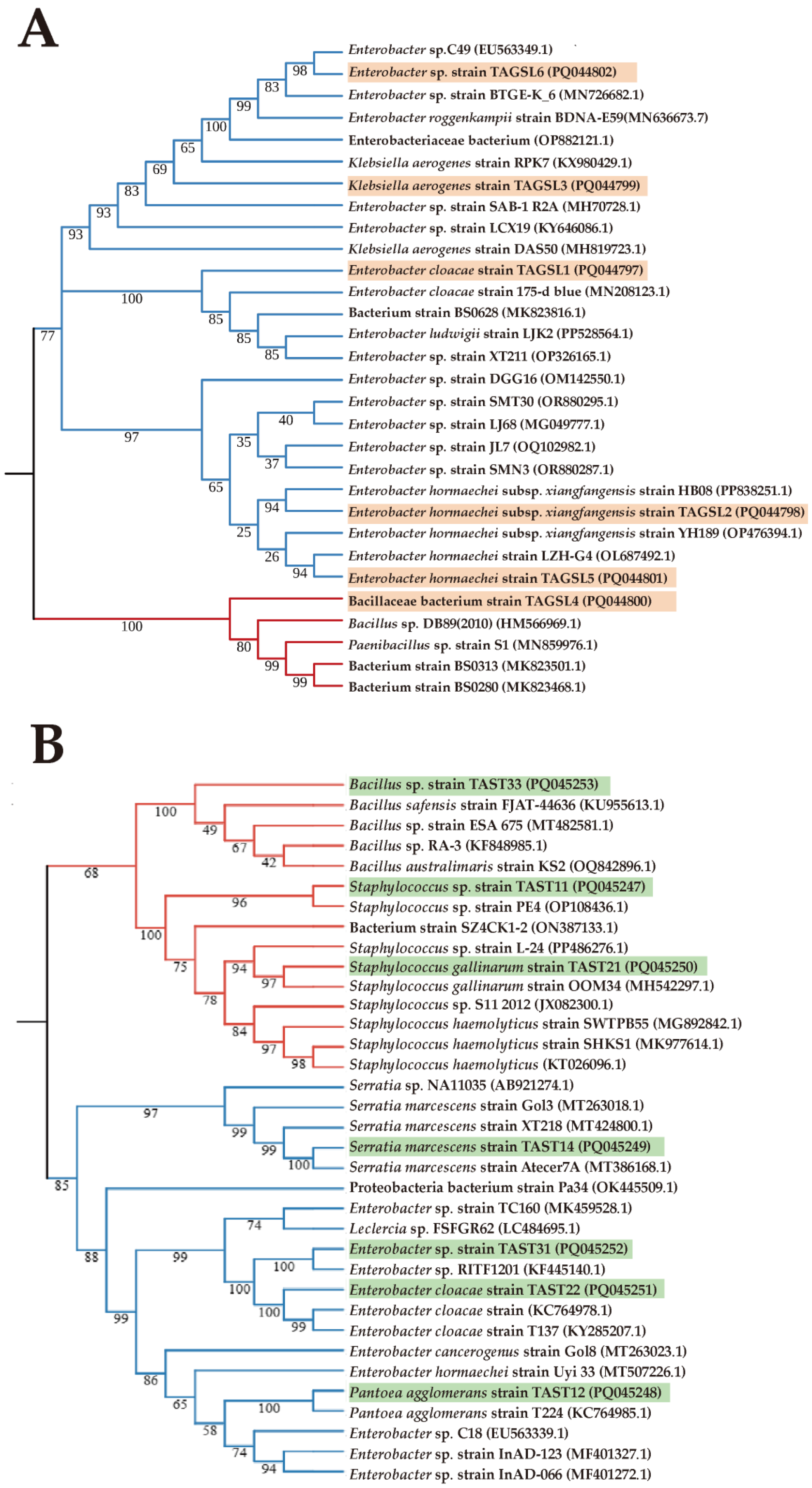

Figure S5. Phylogenetic tree based of gut bacteria of SLTA and STTA: (A) Phylogenetic tree for molecular identification of SLTA gut bacteria isolated in vitro; (B) Phylogenetic tree for molecular identification of STTA gut bacteria isolated in vitro.

Table S1. Statistics on sequencing data of gut samples of SLTA and STTA

Host plants	Sample ID	Raw CCS	Clean CCS	Effective CCS	OUT Number	Sequences Number	Average length (bp)	Effective sequence proportion (%)
Solanum lycopersicum	SLTA1	32,569	32,568	31,991	29	31,875	1,462	98.23
	SLTA2	35,344	35,340	34,803	24	34,650	1,455	98.47
	SLTA3	35,737	35,736	35,121	30	34,888	1,469	98.28
Solanum tuberosum	STTA1	37,304	37,303	36,927	33	36,543	1,465	98.99
	STTA2	27,393	27,389	27,312	32	26,959	1,454	99.70
	STTA3	36,895	36,894	36,859	28	36,635	1,480	99.90

Note: SLTA means *T. absoluta* feeding on tomatoes; STTA means *T. absoluta* feeding on potatoes.

Table S2. Colony morphology and cultural characteristics of bacteria in SLTA and STTA.

Host plants	Strain No.	Cultural characteristics	Cell morphology	Gram stain
Solanum lycopersicum	*Enterobacter cloacae* strain TAGSL1	White translucent, round, smooth and moist	Short rhabditiform	-
	Enterobacter hormaechei subsp. *xiangfangensis* strain TAGSL2	Milky white, round, smooth surface, regular edge	Short rhabditiform	-
	Klebsiella aerogenes strain TAGSL3	White translucent, round, smooth surface, regular edges	Short rhabditiform	-
	Bacillaceae bacterium strain TAGSL4	White transparent, irregular edges, not wet	Rhabditiform	+
	Enterobacter hormaechei strain TAGSL5	White translucent, round, smooth surface, regular edges	Short rhabditiform	-
	Enterobacter sp. strain TAGSL6	White translucent, round, smooth surface, regular edges	Short rhabditiform	-
Solanum tuberosum	*Staphylococcus* sp. strain TAST11	Yellow, round, moist, smooth	Sphericity	+
	Pantoea agglomerans strain TAST12	Milky yellow, round, smooth, irregular edges	Rhabditiform	-
	Serratia marcescens strain TAST14	Round bulge, opaque center, can produce red pigment	Short rhabditiform	-
	Staphylococcus gallinarum strain TAST21	Milky yellow, round, moist, wrinkled at the edges	Sphericity	+
	Enterobacter cloacae strain TAST22	Milky white, round, central protrusion, smooth, wet	Rhabditiform	-
	Enterobacter sp. strain TAST31	White, round, center raised, smooth, wet	Short rhabditiform	-
	Bacillus sp. strain TAST33	Milky white, round, smooth and moist	Spherical rod-shaped	+

Note: "+" represents Gram-positive bacteria; "-" is Gram-negative bacteria.
